# The association of oral microbiota and oral-gut microbial co-presence with obesity in Chinese children

**DOI:** 10.3389/fcimb.2026.1866102

**Published:** 2026-08-25

**Authors:** Qianjin Qi, Chaonan Gao, Yinkun Yan

**Affiliations:** 1 Center for Non-Communicable Disease Management, Beijing Children’s Hospital, Capital Medical University, National Center for Children’s Health, Beijing, China; 2 Department of Epidemiology, Capital Center for Children's Health, Capital Medical University, Capital Institute of Pediatrics, Beijing, China; 3 Postdoctoral Research Station for Public Health and Preventive Medicine, Hebei Medical University, Shijiazhuang, Hebei, China; 4 Department of Epidemiology and Health-Statistics, School of Public Health, Hebei Medical University, Shijiazhuang, Hebei, China; 5 Hebei Key Laboratory of Environment and Human Health, Hebei Medical University, Shijiazhuang, Hebei, China

**Keywords:** childhood obesity, Chinese children, gut microbiota, oral microbiota, oral-gut microbial co-presence

## Abstract

**Aim:**

The oral cavity harbors a complex microbial community that is increasingly recognized for its association with systemic diseases, such as cardiovascular diseases. Furthermore, a significant proportion of oral microbiota is swallowed, potentially influencing the gut microbiota. This potential oral-gut microbial linkage is hypothesized as a pathway through which oral microbes exert systemic effects. This study aims to investigate the association between oral microbiota and childhood obesity, and to explore the co-presence patterns between oral and gut microbiota in relation to childhood obesity.

**Materials and methods:**

This study is a case-control study. We recruited participants aged 6–17 years from Beijing Children’s Hospital, collecting saliva and fecal samples. Both parts of the study performed 16S rRNA gene sequencing on the samples, comparing microbial diversity between obese (OB) and normal-weight (NW) children. We aggregated relative abundances of major bacterial phyla and genera to identify differentially abundant genera between groups. Linear discriminant analysis effect size (LEfSe) was used to identify genera significantly different between NW and OB children. Additionally, we examined correlations between key oral genera and clinical indicators. Genera detected in both oral cavity and gut samples at > 10% prevalence were defined as “co-present genera”. A random forest model was constructed to distinguish NW from OB children.

**Results:**

A total of 136 children were enrolled, comprising 68 individuals in the OB group and 68 in the NW group. There were no significant differences observed in age, gender, birth weight, or height. Oral microbiota diversity and composition significantly differed between OB and NW groups. LEfSe analysis identified *Neisseria*, *Peptostreptococcus*, and *Oribacterium* as potential discriminative genera in the OB oral microbiota. The co-present genera exhibited distinct relative abundance patterns across sites. A Random Forest model based on 23 oral and 7 gut co-present genera achieved an area under the curve of 0.73. Notably, both *Veillonella* and *Bifidobacterium* were identified as key bacterial genera in both the oral cavity and the gut.

**Conclusions:**

This study indicated that the oral microbiome is closely associated with childhood obesity. Consequently, the oral microbiome represents a highly attractive target for future obesity prevention strategies.

## Introduction

Obesity has already become a critical public health challenge globally, especially in children. Compared to 2000 to 2011, a 1.5-fold increase in the prevalence of obesity was observed in 2012 to 2023 (Zhang et al., 2024). Childhood obesity tends to persist through adulthood, being associated with diabetes, cancer, and cardiometabolic outcomes in midlife (Horesh et al., 2021). Meanwhile, effective interventions for childhood obesity remain inadequate. Obesity is influenced not only by genetic and environmental factors but also by microorganisms (Zhou et al., 2023). Multi-omics-based systematic analyses have shown that dysbiosis of intestinal flora is associated with simple obesity in children (Zhang et al., 2015). A recent randomized controlled trial of fecal microbiota transplantation (FMT) in adolescents with obesity demonstrated sustained improvements in metabolic health and systemic inflammation at 4-year follow-up, with long-term alterations in gut microbiome composition and function (Wilson et al., 2025). Oral microbiota composition is regulated by host and environmental factors, and its disruption is closely linked to health and systemic diseases (Peng et al., 2022). Oral microbial diversity and composition exhibit significant alterations in obese children, and specific bacteria are enriched (Balakrishnan et al., 2021), whereas the relative abundance of *Veillonella*, *Prevotella*, and *Streptococcus* is reduced (Raju et al., 2019). However, microbiome composition is strongly shaped by host genetics, diet, and lifestyle, and whether these findings are generalizable to Chinese pediatric populations remains unknown, which motivates our study in a Beijing-based cohort.

The oral and gut, as the two ends of the digestive tract, do not exist independently of each other (Kunath et al., 2024). Despite oral microbes being swallowed in large quantities every day, they are often difficult to colonize a healthy gut due to the colonization resistance of the resident gut bacteria (Baker et al., 2024). However, under certain conditions, such as gut microbial dysbiosis or exposure to some drugs, oral microbes may break through colonization resistance, leading to an increase in their abundance in the gut (Xiao et al., 2024; Zhu et al., 2024). Salivary microbiota flow will induce gut microbiota dysbiosis, suggesting the potential impact of oral microbiota translocation in disease development (Bao et al., 2022). Recent evidence indicates that imbalances in gut and oral microbiota are linked to obesity, with niche-specific microbial profiles influencing metabolic dysfunction and weight gain (Hu et al., 2025). However, it remains unclear whether oral and gut microbial communities are ecologically associated with childhood obesity.

We therefore hypothesized that obese children harbor distinct oral and gut microbial compositions compared to normal-weight controls, and that the similarity between oral and gut microbial communities is altered in obesity. This research will provide a new perspective on childhood obesity and provide a theoretical basis for the future development of microbiota-based early intervention and personalized treatment strategies.

## Methods

### Study design and participants

This study is a case-control study based on a clinical cohort. Participants were recruited between January 2025 and December 2025 at the Beijing Children’s Hospital. Participants were aged between 6–17 years old and were able to cooperate in completing body measurements, information collection, and sample collection. The exclusion criteria were 1) use of antibiotics or microbiological agents in the last 2 months, 2) abnormal frequency of stools (less than three times per week or more than three times per day), 3) gastrointestinal diseases (e.g., ulcerative colitis, Crohn’s disease, or acute diarrhea) and oral diseases (e.g., periodontitis, dental caries), 4) major illnesses (e.g., infectious diseases, psychiatric disorders, and malignant neoplasms), and 5) Children with secondary obesity (e.g. Cushing’s syndrome, hypothyroidism, etc.). This study was approved by the Ethics Committee of Beijing Children’s Hospital (2024-Y-256-D). Participants older than 8 years old and their parents provided written informed consent, and participants younger than 8 years old were provided written informed consent by their parents.

A pilot study was conducted before the main study. After matching participants 1:1 by age and gender, 8 participants were enrolled in each of the obese and normal-weight groups. With a Type I error set at 0.05 (two-tailed) and a Type II error set at 0.1, the calculated sample size was 66 participants per group.

Height and weight were measured by a physician using an ultrasonic height and weight meter (HNH-219, Omron, Kyoto, Japan). Body mass index (BMI) was calculated as weight in kilograms divided by height in meters squared. Obesity was diagnosed according to the Chinese screening for overweight and obesity among school-age children and adolescents (WS/T 586-2018, **Supplementary Methods 1**). Participants’ information was collected through a questionnaire completed by parents. Measuring methods for other clinical parameters are described in the **Supplementary Methods 2**.

### Saliva and fecal sample DNA extraction and amplicon sequencing

Saliva and fecal samples were collected by following the standard sampling procedures of the Human Microbiome Project ("Human Microbiome Project Standard Operating Procedures"). For the detailed collection process, refer to the **Supplementary Methods 3**. DNA was extracted from collected fecal samples using the QIAamp DNA Mini Kit (Qiagen, Valencia, CA, USA) according to the manufacturer’s protocols. For saliva samples, DNA was extracted using Proteinase K cleavage. The quality of the DNA was examined by electrophoresis on a 1.2% agarose gel. Using amplicon sequencing targeting the highly variable region V3-V4 of the bacterial 16S rRNA gene, primers 357F (5’-ACTCCTACGGRAGGCAGCAG-3’) and 806R (5’-GGACTACHVGGGTWTCTAAT-3’) were used. Library construction and sequencing were performed using NovaSeq 6000 (Illumina, San Diego, CA, USA).

### Sequencing data processing and taxonomic assignment

The raw data obtained from sequencing were split by barcode to obtain valid sequences for each sample. Low-quality sequences were removed using Trimmomatic (0.38), and sequencing junctions and primers were processed using Cutadapt (1.16). The sequences were spliced according to the overlap relationship using Flash (1.2.11), while Mothur (1.33.3) was used for quality control and filtering. The optimized sequences were obtained by removing the sequences containing ambiguous bases, single-base highly repetitive regions (homologous=8), excessively long (max length> 485 bp) and short (min length< 200 bp) sequences, as well as the chimeras generated in the Polymerase Chain Reaction process.

The optimized sequences were subjected to Amplicon Sequence Variant (ASV) analysis and species taxonomic annotation. ASV clustering was performed using DADA2 in Qiime2 (2020.8.0), and singleton ASVs (ASVs that occur only once) were removed. Subsequently, the ASV representative sequences were annotated to species (Silva 128 database). Based on the annotation results, species abundance tables at phylum, class, order, family, genus, and species levels were generated and further analyzed for diversity in community structure.

### Statistical analysis

To ensure comparability between groups, this study employed propensity score matching (PSM). Participants in the NW group and the OB group were matched 1:1 using the nearest-neighbor matching algorithm based on age and sex. Continuous variables were expressed as means (standard errors) and compared between groups using the T-test, and categorical variables were expressed as frequencies (percentages) and compared between groups using chi-square tests. Alpha diversity was assessed using the Shannon index and observed ASVs. The significance of differences between groups was analyzed using the Wilcoxon rank sum test. Principal Coordinate Analysis (PCoA) and Permutational Multivariate Analysis of Variance (PERMANOVA) were performed to test for differences in beta diversity based on the Bray-Curtis covariance matrix.

To further explore differences in the composition of the microbiota, the relative abundance was sorted, extracting the top 10 phylum-level classifications and the top 20 genus-level classifications with the highest abundance, and categorizing the rest of the species as “Others”. Using the Wilcoxon signed-rank test and correcting the *P*-value via the Benjamini-Hochberg (BH) method, we performed difference tests at the corresponding level. Prevalence is used to reflect the distribution of microorganisms in a population. Oral/gut bacteria refer to bacteria observed in 10% or more of saliva/fecal samples, based on the criteria used in previous studies (Schmidt et al., 2019). Genera with prevalence > 10% in both saliva and fecal samples were defined as co-present genera. Exclude genera with average relative abundance < 0.01% or prevalence < 10%. Linear discriminant analysis effect size (LEfSe) was used to identify bacteria with significant differences between the obese and normal weight groups. The Sparse Correlations for Compositional Data (sparCC) was calculated using the “SpiecEasi” R-package and corrected for False Discovery Rate (FDR), and a heatmap was plotted using the “Pheatmap” R-package. We predicted the functional potential (Kyoto Encyclopedia of Genes and Genomes, KEGG) of the microbial communities from 16S rRNA gene sequencing data using Phylogenetic Investigation of Communities by Reconstruction of Unobserved States 2 (PICRUSt2).

Among the co-present genera, 23 oral and 7 gut genera were selected for random forest model construction based on feature importance assessed by the mean decrease in Gini index, with a threshold of Gini index > 0.6. A random forest model was trained using 10-times repeated 10-fold cross-validation to reduce variance associated with random data partitioning, with feature importance assessed by the Gini index. Model performance was evaluated using Area Under the Curve (AUC) of Receiver Operating Characteristic (ROC), sensitivity, specificity, accuracy, precision, and F1 score (**Supplementary Methods 4**). To assess model convergence, the Out-of-Bag (OOB) error was recorded during random forest training (ntree = 500, mtry = optimal value), and a convergence curve was plotted. All analyses were performed using R (4.2.1, R package versions used in this study describe in **Supplementary Methods 5**). Unless otherwise stated, all statistical tests were two-sided.

## Results

### Characteristics of study participants

A total of 136 participants were finally recruited, including 68 OB children and 68 NW children (**Table 1**). There were no significant differences between groups for age, birth weight, and sex (*P* > 0.05). The OB group had significantly higher BMI (26.98 ± 4.29 vs. 17.44 ± 2.35 kg/m², *P* < 0.001) and waist-to-hip ratio (0.93 ± 0.05 vs. 0.83 ± 0.08, *P* < 0.001) than the NW group.

**Table 1:** Characteristics of the participants.

Variable	Total n = 136	NW n = 68	OB n = 68	*P* value
Age, years	10.61 ± 2.74	10.95 ± 2.93	10.27 ± 2.52	0.15
Sex				0.39
Boy	74 (54.41)	34 (50)	40 (58.82)	
Girl	62 (45.59)	34 (50)	28 (41.18)	
Height, cm	148.54 ± 15.66	147.21 ± 16.74	149.87 ± 14.5	0.32
Weight, kg	50.64 ± 20.02	39.11 ± 12.77	62.16 ± 19.36	< 0.01
BMI, kg/m^2^	22.21 ± 5.89	17.44 ± 2.35	26.98 ± 4.29	< 0.01
Waist circumference, cm	72.69 ± 17.72	60.57 ± 11.38	84.8 ± 14.33	< 0.01
Hip circumference, cm	82.34 ± 16.85	73 ± 12.74	91.69 ± 15.25	< 0.01
Waist hip ratio	0.88 ± 0.08	0.83 ± 0.08	0.93 ± 0.05	< 0.01
Birth weight, kg	3.39 ± 0.53	3.33 ± 0.52	3.47 ± 0.53	0.12

Continuous data are presented as mean ± standard error, and categorical data are presented as frequency (percentage).

### Oral and gut microbiota diversity and composition

**Figure 1:**
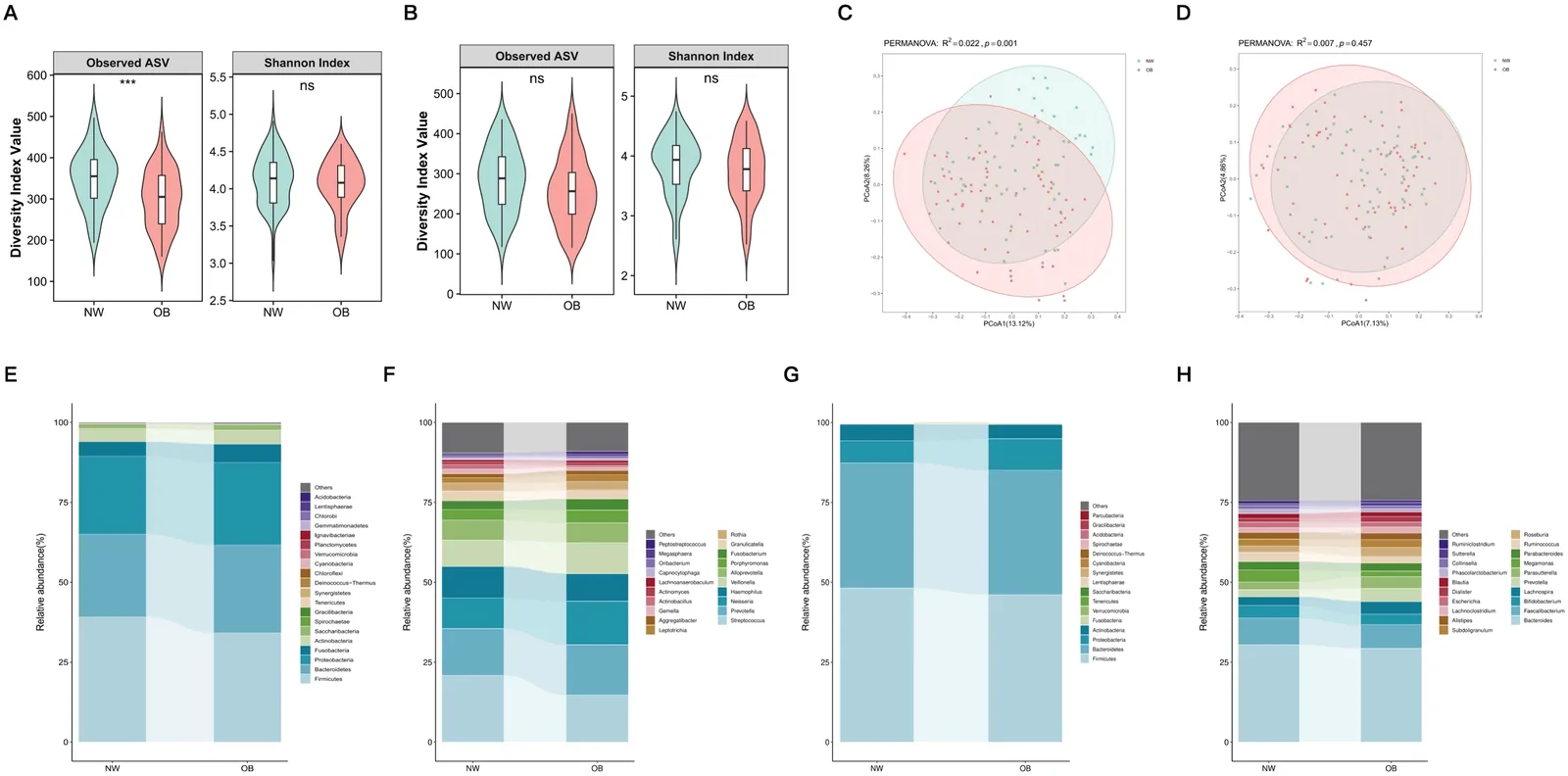
Differences in oral and gut microbial diversity and composition between obese and normal weight children. **(A, B)** Observed ASV and Shannon index of oral **(A)** and gut **(B)** microbiota. Boxplots show mediums; the lower and upper hinges correspond to the first and third quartiles (the 25th and 75th percentiles); whiskers extend to the largest and smallest values at 1.5*IQR (interquartile range) from the hinge. *p < 0.05, **p < 0.01, ***p < 0.001; ns, no significance. **(C, D)** Principal coordinate analysis (PCoA) based on Bray-Curtis dissimilarities of oral **(C)** and gut **(D)** microbiota, colored by group. Explained variance (R²) and P values were calculated using PERMANOVA tests. **(E-H)** Mosaic plot of the proportion of participants in the oral microbial clusters at the phylum level **(E)** and genus level **(F)**. Mosaic plot of the proportion of participants in the gut microbial clusters at the phylum level **(G)** and genus level **(H)**. NW, normal weight; OB, obesity.

We identified 20 phyla and 253 genera in the saliva and 16 phyla and 232 genera in feces. The rarefaction curve indicates the current sequencing volume is sufficient to reflect the true diversity of the sample (**Supplementary Figures 1A, C**). Compared with NW group, OB group had fewer observed ASVs and lower alpha diversity indices in oral, but there were no significant differences in gut (**Figures 1A, B**; **Supplementary Figures 1B, D**). There were significant differences in the composition of oral microbiota between OB and NW groups (**Figure 1C**, PERMANOVA *P* = 0.001, R^2^ = 0.222), but not in the composition of the gut microbiota (**Figure 1D**, PERMANOVA *P* = 0.457, R^2^ = 0.007).

**Figure 2:**
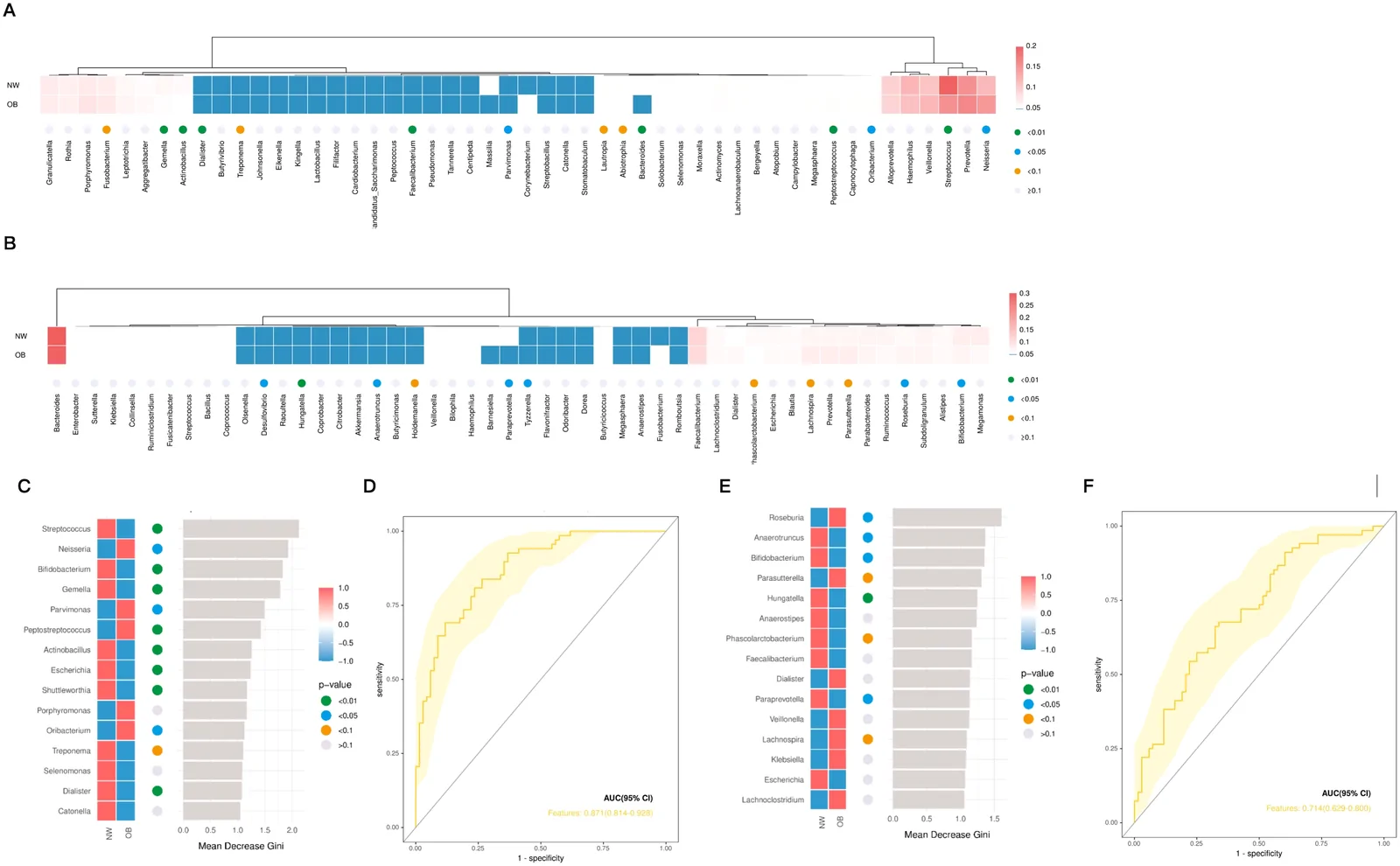
Microbiome–based discrimination of childhood obesity. **(A, B)** Clustering heatmaps showing relative abundances of the top 50 genera in the oral **(A)** and gut **(B)**. **(C, E)** Random Forest regression analysis of oral **(C)** and gut **(E)** microbiota at genus level. The heatmap represents relative abundances of microbes. **(D, F)** Performance of the Random Forest classifier to distinguish the oral **(D)** and gut **(F)** microbial profiles between obesity and normal weight children. AUC, area under curve; NW, normal weight; OB, obesity.

Firmicutes, Bacteroidetes, and Proteobacteria are the major phyla in oral, while Firmicutes and Bacteroidetes are the major phyla in gut (**Figures 1E, G**, relative abundance >10%). Compared with NW group, OB group showed a significant reduction in Firmicutes in oral (**Supplementary Table 1**, *FDR* < 0.05). Compared with NW group, the OB group had significantly higher relative abundance of *Streptococcus*, *Gemella*, and *Actinobacillus* in oral, and significantly lower abundance of *Peptostreptococcus* (**Figures 1F, H**; **Supplementary Table 2**, *FDR* < 0.05). Among the top 50 genera in relative abundance, 14 genera in oral and 11 genera in the gut showed significant differences between NW and OB groups (**Figures 2A, B**, *P* < 0.1). To investigate the effectiveness of oral and gut microbiota in distinguishing between OB and NW groups, we constructed a random forest classifier. Based on the Gini index, we screened the top 15 important oral and gut genera (**Figures 2C, E**). The AUC for the oral and gut microbiota were 0.87 (0.81-0.93) and 0.71 (0.63-0.80), respectively (**Figures 2D, F**). The oral and gut random forest models were built using different feature sets. Therefore, their AUC values (0.87 and 0.71) are not directly comparable, and no statistical test was performed between the two models.

### Altered microbial metabolic pathways in obese children

**Figure 3:**
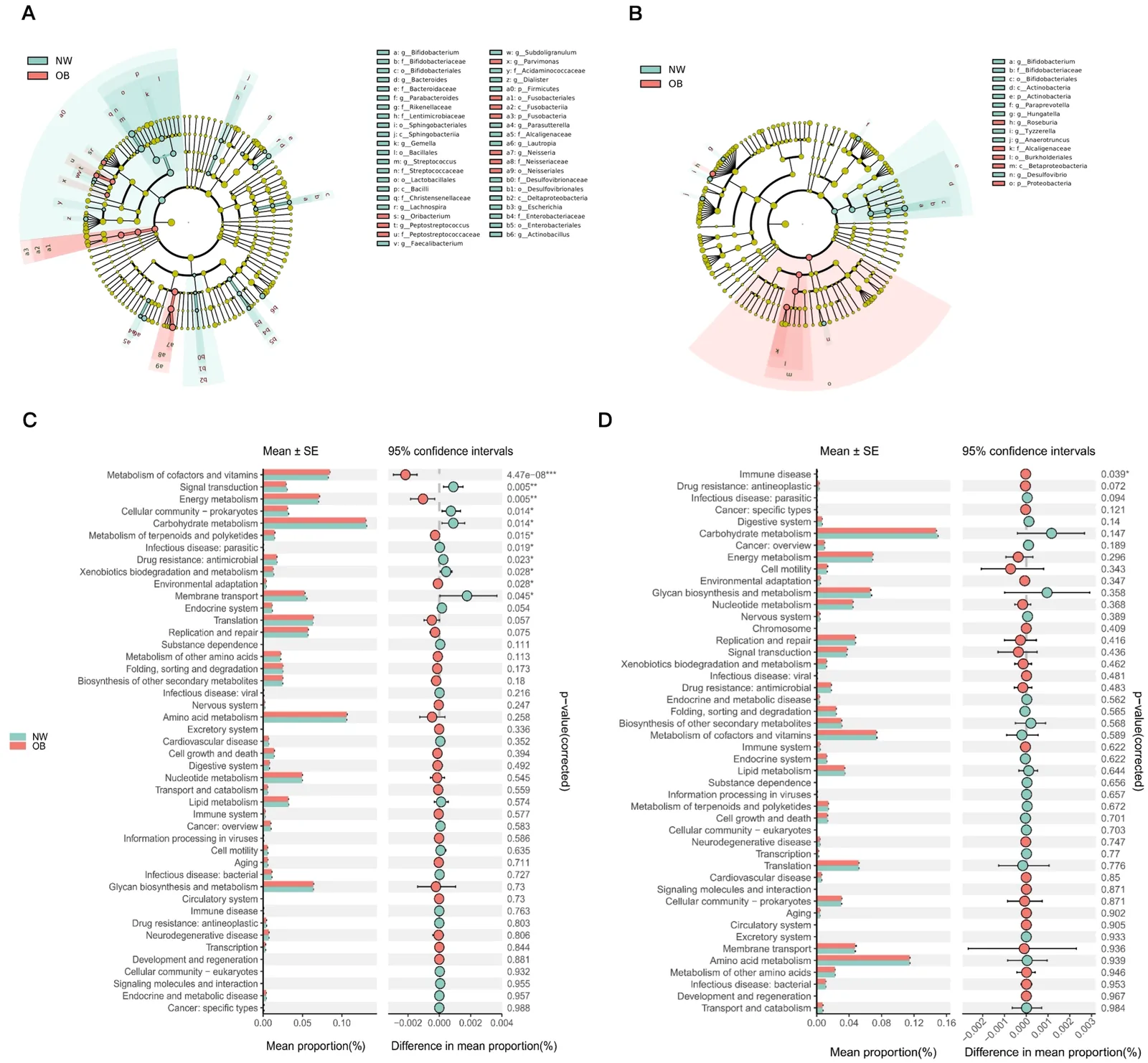
Oral and gut microbial biomarkers and functional pathways associated with childhood obesity. **(A, B)** Linear discriminant analysis (LDA) effect size showed a list of specific oral **(A)** and gut **(B)** microbiota that enable discrimination between obesity and normal weight children. Genera enriched in obesity group are indicated with a positive LDA score (red), and genera enriched in normal weight group have a negative score (green). **(C, D)** Comparison of KEGG pathways at level 2 in the oral **(C)** and gut **(D)** Microbiota. NW, normal weight; OB, obesity; g, genus. KEGG, The Kyoto Encyclopedia of Genes and Genomes; SE, Standard Error. *p < 0.05, **p < 0.01, ***p < 0.001.

Furthermore, we screened for genera with significant differences between groups (**Supplementary Figure 3**). LEfSe analysis revealed that *Neisseria*, *Peptostreptococcus*, and *Oribacterium* in oral, along with *Roseburia* in gut, were the top discriminative taxa for the OB group, while *Streptococcus* (oral) and *Bifidobacterium* (gut) were the top discriminative taxa for the NW group (**Figures 3A, B**). We further analyzed potential changes in microbial functions using the KEGG database. For oral microbiota, we obtained 6 primary pathways, 45 secondary pathways, and 365 tertiary pathways; for gut microbiota, we obtained 6 primary pathways, 46 secondary pathways, and 363 tertiary pathways (primary pathways in **Supplementary Figure 4**). OB group had significantly increased activity in pathways such as metabolism of cofactors and vitamins (*P* < 0.001), energy metabolism (*P* = 0.005), metabolism of terpenoids and polyketides (*P* = 0.015), and environmental adaptation (*P* = 0.028) in oral (**Figure 3C**). Meanwhile, the activity of pathways such as immune disease (*P* = 0.039) in gut microbiota was significantly increased (**Figure 3D**).

### Associations between oral/gut microbiota and BMI and other clinical parameters

**Figure 4:**
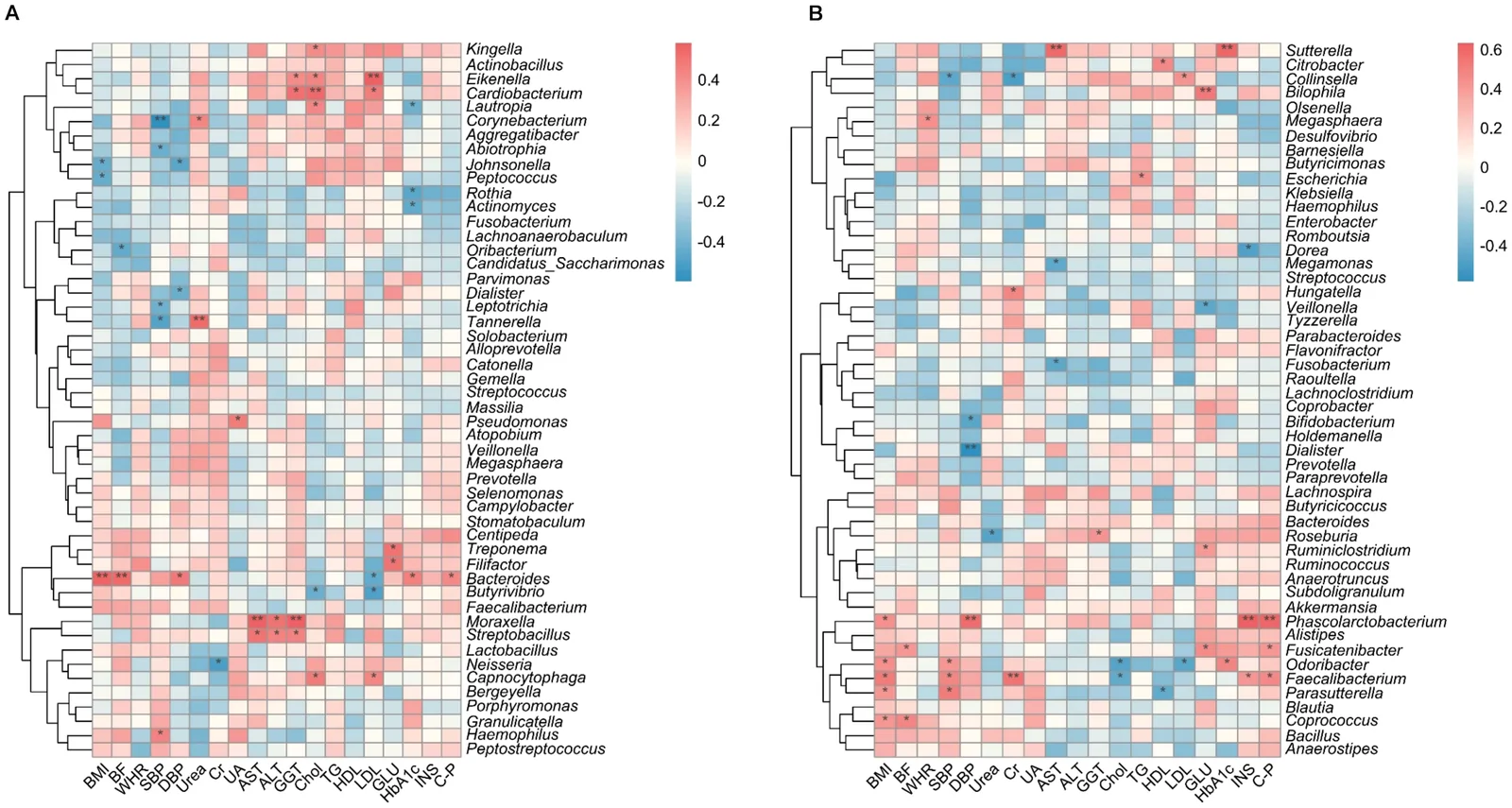
The oral and gut microbiota are closely related to children's BMI and other clinical parameters. Heatmaps of Spearman’s correlation coefficients between clinical parameters and relative abundances of the top 50 genera in microbiota of oral **(A)** and gut **(B)**. BMI, Body Mass Index; BF, Body Fat Percentage; WHR, Waist Hip Ratio; SBP, Systolic Blood Pressure; DBP, Diastolic Blood Pressure; Cr, Creatinine; UA, Uric Acid; AST, Aspartate Aminotransferase; ALT, Alanine Aminotransferase; GGT, Gamma-glutamyl Transferase; Chol, Cholesterol; TG, Triglycerides; HDL, High-Density Lipoprotein; LDL, Low-Density Lipoprotein; GLU, Glucose; HbA1c, Glycated Hemoglobin; INS, Insulin; C-P, Connecting Peptide. *p < 0.05, **p < 0.01, ***p < 0.001.

We evaluated the association between BMI and clinical parameters with the relative abundance of the top 50 genera in saliva and feces. BMI was significantly positively correlated with *Bacteroides* in oral and negatively correlated with *Johnsonella* and *Peptococcus* (**Figure 4A**). Notably, the relative abundance of *Bacteroides* was also positively correlated with body fat percentage, diastolic blood pressure, glycated hemoglobin, and C-peptide. BMI was significantly positively correlated with *Faecalibacterium*, *Coprococcus*, *Parasutterella*, *Odoribacter*, and *Phascolarctobacterium* in the gut (**Figure 4B**). Among these, *Faecalibacterium* was also positively correlated with systolic blood pressure, creatinine, insulin, and C-peptide. In summary, these results indicate that the oral/gut microbiota is closely related to BMI and clinical parameters.

### Oral-gut microbial co-presence is associated with childhood obesity

**Figure 5:**
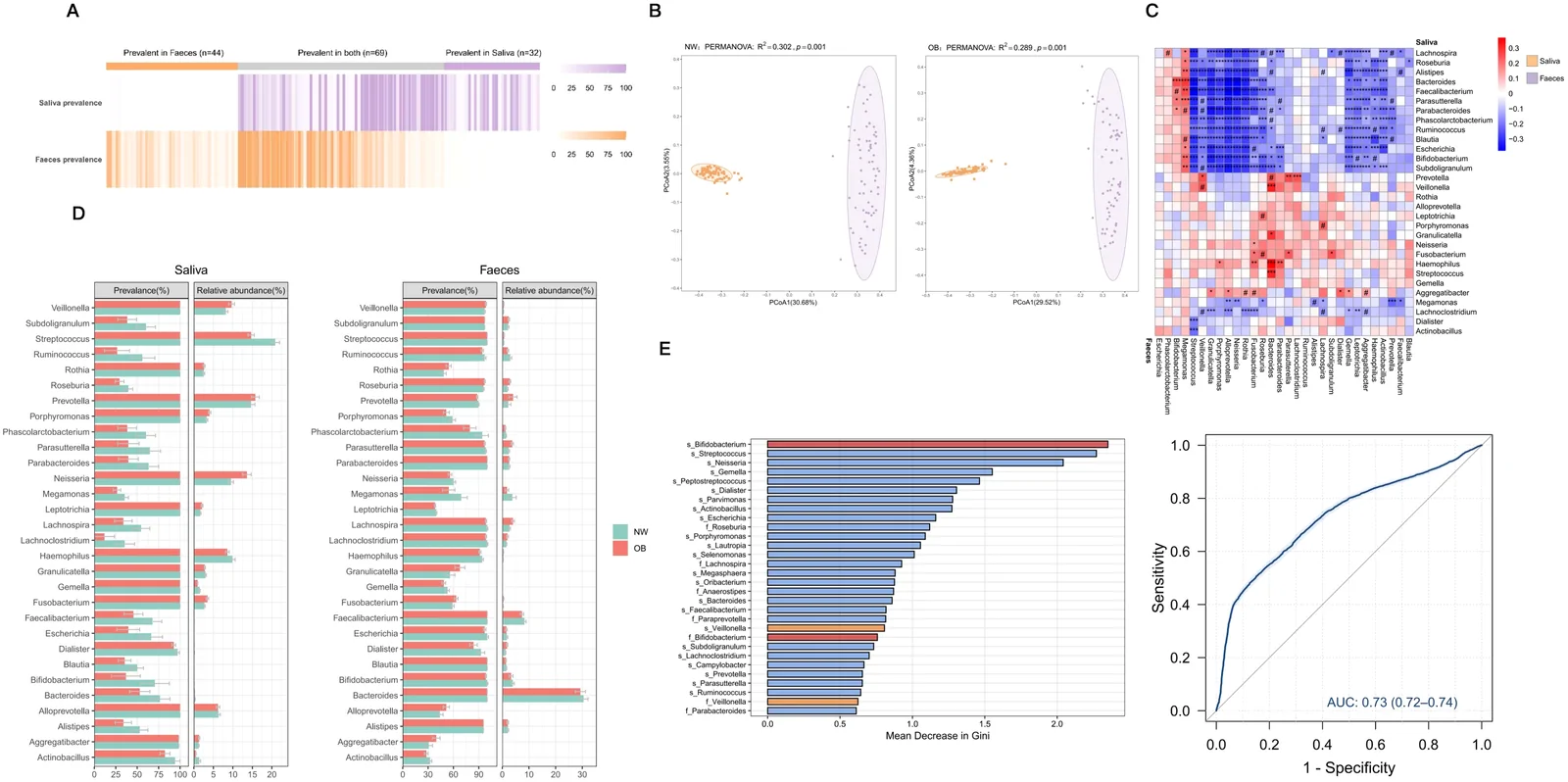
Communication between oral and gut microbiota in obese and normal weight children. **(A)** Overview of genera commonly present in the oral and gut. **(B)** Principal coordinate analysis (PCoA) based on Bray-Curtis dissimilarities, colored by position. Explained variance (R^2^) and *P* values were calculated using PERMANOVA tests. **(C)** Heatmaps of Sparse Correlations for Compositional among relative abundances of the top 30 shared genera between the oral and the gut. # FDR < 0.1, * FDR < 0.05, ** FDR < 0.01, *** FDR < 0.001. **(D)** Prevalence and relative abundances of the top 30 shared genera in oral and gut. **(E)** Performance of the Random Forest classifier to distinguish the Oral cavity-gut shared microbial profiles between obesity and normal weight children. s_, saliva; f_, feces. NW, normal weight; OB, obesity.

We expanded our analysis to investigate the co-presence patterns between oral and gut microbiota in OB. We identified 69 shared genera with a prevalence > 10% in both oral and gut (**Figure 5A**). The microbial composition of the oral and gut differed significantly (**Supplementary Figure 5**). The ecological position explanatory power (R^2^) for the NW group and OB group was 30.2% and 28.9%, respectively, indicating reduced microbial ecological position specialization between the oral and gut in obese children (**Figure 5B**). The relative abundances of *Veillonella*, *Fusobacterium*, and *Aggregatibacter* in the gut were positively correlated with those in the oral cavity, whereas *Parabacteroides* exhibited a negative correlation between these two sites (**Figure 5C**). We further performed correlation analysis on the top 30 genera in relative abundance in the oral cavity and gut. We found that *Streptococcus* and *Gemella* were strongly positively correlated in both the gut and oral (**Supplementary Figure 6**). The relative abundance and prevalence of the top 30 co-present genera indicate a reciprocal relationship between oral and gut (**Figure 5D**). Genera such as *Streptococcus* and *Gemella*, which are commonly found in the oral, were more prevalent or abundant in the gut of the OB group than in the NW group (**Supplementary Figure 7**). Furthermore, 23 oral and 7 gut genera were identified from the 69 co-present genera based on Gini index (**Supplementary Table 3**). A random forest model based on these genera demonstrated high predictive performance, achieving an AUC of 0.73 (95% CI: 0.72-0.74) (**Figure 5E**). We compared the combined model with oral-only (AUC = 0.78) and gut-only (AUC = 0.55) sub-models (**Supplementary Figure 8**). Oral genera have captured most of the predicted signals, while gut genera have not provided any additional gain. The out-of-bag error was monitored during tree construction to assess model convergence (**Supplementary Figure 9**). Notably, both *Veillonella* and *Bifidobacterium* were identified as key bacterial genera in both the oral cavity and the gut. In summary, these findings suggest that childhood obesity is closely associated with oral-gut microbial co-presence patterns.

## Discussion

Our study revealed differences in the diversity and composition of the oral and gut microbiota between normal-weight and obese children. Furthermore, we identified specific oral-gut co-presence genera that are associated with childhood obesity.

Many studies have shown that the diversity and composition of the salivary microbiota are significantly associated with childhood obesity. Zeigler et al. found that obese adolescents had 23 types of bacteria in their oral cavities compared to normal-weight adolescents, with *Neisseria mucosa* being six times higher (Zeigler et al., 2012). Furthermore, metabolic syndrome and obese adolescents show a reduction in health-related species, an increase in inflammatory cytokines and disease-related metabolites in saliva, as well as elevated levels of leptin and reduced levels of adiponectin in saliva (Salman et al., 2025). Even as early as infancy, the oral microbiota of children who gain weight rapidly already shows patterns commonly seen in obese adults (Craig et al., 2018). This study found that *Bifidobacterium* and *Veillonella* are genera found in both the oral and gut microbiomes and are closely associated with childhood obesity. Silva et al. found that the abundance of *Bifidobacterium* in the gut was significantly lower in obese children compared to normal-weight children (Da Silva et al., 2020). Chen et al. identified *Veillonella* as a frequent oral-gut transmitter capable of ectopic colonization in the gut, supporting our observation of its co−presence between the two sites (Chen et al., 2023).

Several lines of evidence support the biological plausibility of ecological linkages between oral and gut microbiota. Oral pathogens may cause inflammatory responses and induce the intestinal microenvironment to transition from a healthy state to a pre-disease state (Lu et al., 2023). There are currently two hypotheses regarding the mechanism behind the increase in the relative abundance of oral bacteria in feces: the expansion hypothesis suggests that oral bacteria proliferate and expand in the intestine; the marker hypothesis suggests that the increase reflects the depletion of intestinal bacteria and is a marker of changes in the intestinal microbiota (Liao et al., 2024). After administering periodontitis patient saliva orally or via gavage to mice, abnormal enrichment of oral pathogens was detected in the intestines (Atarashi et al., 2017; Bao et al., 2022). Oral bacteria are unlikely to be found in the distal intestine of healthy adults (Rashidi et al., 2024). Cheung et al. also found similar results, that the oral-intestinal transmission of bacterial and fungal microbiota in healthy adults is limited, and the sharing of potentially transmissible fungal ASVs is associated with a higher BMI (Cheung et al., 2023). Our study in obese children revealed altered ecological coordination between oral and gut microbiota. The contrast implies that obesity-related metabolic or inflammatory states may disrupt the normal segregation between oral and gut microbial communities, potentially facilitating greater microbial ecological association.

The oral microbiome may contribute to obesity through several mechanisms. Firstly, the oral microbiota has a certain influence on taste perception thresholds, which in turn affects health outcomes (such as obesity and dental caries) through dietary habits. Numerous studies have demonstrated significant differences in salivary biochemical variables and specific bacterial communities between obese and normal-weight groups, some of which are significantly associated with taste perception (Lopez-Davalos et al., 2023; Mameli et al., 2019). Especially, sweet taste perception is associated with the oral microbial metabolism of sugar (Gardner et al., 2020). Secondly, oral pathogens may also influence inflammation levels. Oral dysbiosis and periodontal disease are associated with systemic inflammation in populations (Wang et al., 2019). This link is supported by animal models of periodontitis, in which oral microbiota dysbiosis exacerbates obesity-related inflammation in adipose tissue and the liver by increasing macrophage invasion (Endo et al., 2010). Finally, oral bacteria colonizing the gut can trigger inflammation by disrupting gut microbiota, altering T helper cell balance, and increasing M1/M2 macrophage ratios (Kobayashi et al., 2020; Cox et al., 2015; Atarashi et al., 2017). Consistent with this mechanism, disruption of the oral microbiota in Western-fed mice reduces fat accumulation by affecting the gut microbiota (Carvalho et al., 2024). Although direct mechanistic evidence for the genera we identified is still limited, the above pathways support a plausible role of the oral microbiome in childhood obesity.

There are still some limitations. Firstly, due to the case-control study design, we cannot establish causality between the microbiome and childhood obesity. Secondly, 16S rRNA gene sequencing targeting the V3-V4 region limited resolution to the genus level and cannot support strain-level transmission claims. Future studies using full-length 16S or shotgun metagenomic sequencing could further validate our findings at the species or strain level. Third, DNA extraction protocols differed between sample types: the QIAamp DNA Mini Kit was used for fecal samples, whereas a Proteinase K digestion method was used for saliva samples. These two methods may differ in lysis efficiency. Furthermore, although we used PSM to control for age and sex, other potential confounders such as dietary patterns and socioeconomic status were not fully captured. While active caries and periodontitis were excluded, subclinical oral conditions or variations in plaque accumulation were not specifically quantified. Additionally, we did not account for dietary patterns or the use of proton pump inhibitors or other medications that may influence oral-gut microbial transmission. We also acknowledge that PICRUSt2-based functional predictions are less accurate for oral than for gut microbiomes due to reference genome bias, so these results should be interpreted as exploratory rather than definitive. Finally, as the clinical cohort was derived from a single center, the random forest model lacks independent external validation in a separate cohort; therefore, the generalizability of our findings remains to be confirmed.

## Conclusions

The oral and gut microbiota, as well as oral-gut microbial co-presence patterns, are closely associated with childhood obesity. Interventions targeting the oral microbiome may serve as an effective strategy for the prevention and treatment of childhood obesity.

## Data Availability

The datasets presented in this study can be found in online repositories. The names of the repository/repositories and accession number(s) can be found below: https://ngdc.cncb.ac.cn/bioproject/browse/PRJCA046679, PRJCA046679.
