# Supplementary material for: The association of oral microbiota and oral-gut microbial co-presence with obesity in Chinese children
Source: Front Cell Infect Microbiol. 2026 Aug 25;16:1866102. doi: 10.3389/fcimb.2026.1866102 (PMC13549852; doi:10.3389/fcimb.2026.1866102)
Supplement: Supplementary file 1 [file DataSheet1.docx]

**Supplement** **method 1.** Comparison of BMI cut‑offs for overweight and obesity in Chinese children and adolescents (WS/T 586‑2018) with WHO and IOTF reference standards

| **Age (years)** | **Sex** | **WS/T 586-2018 (China)**  **Overweight / Obesity (kg/m²)** | **WHO 2007**  **Overweight / Obesity (kg/m²)** | **IOTF 2000**  **Overweight / Obesity (kg/m²)** |
| --- | --- | --- | --- | --- |
| 6.0 | Boys | 16.4 / 17.7 | 16.8 / 18.1 | 17.3 / 18.8 |
|  | Girls | 16.2 / 17.5 | 16.6 / 17.9 | 17.1 / 18.6 |
| 6.5 | Boys | 16.7 / 18.1 | 17.0 / 18.3 | 17.5 / 19.1 |
|  | Girls | 16.5 / 17.9 | 16.8 / 18.2 | 17.4 / 18.9 |
| 7.0 | Boys | 17.0 / 18.5 | 17.2 / 18.6 | 17.8 / 19.5 |
|  | Girls | 16.8 / 18.3 | 17.0 / 18.5 | 17.7 / 19.2 |
| 7.5 | Boys | 17.4 / 19.0 | 17.5 / 19.0 | 18.2 / 20.0 |
|  | Girls | 17.2 / 18.8 | 17.3 / 18.8 | 18.1 / 19.8 |
| 8.0 | Boys | 17.8 / 19.7 | 17.8 / 19.5 | 18.8 / 21.0 |
|  | Girls | 17.6 / 19.4 | 17.6 / 19.2 | 18.7 / 20.8 |
| 8.5 | Boys | 18.3 / 20.3 | 18.2 / 20.0 | 19.2 / 21.5 |
|  | Girls | 18.1 / 20.0 | 18.0 / 19.8 | 19.1 / 21.4 |
| 9.0 | Boys | 18.8 / 21.0 | 18.6 / 20.5 | 19.6 / 22.0 |
|  | Girls | 18.7 / 20.7 | 18.5 / 20.4 | 19.5 / 22.0 |
| 9.5 | Boys | 19.4 / 21.6 | 19.1 / 21.0 | 20.0 / 22.4 |
|  | Girls | 19.4 / 21.3 | 19.0 / 21.0 | 20.0 / 22.4 |
| 10.0 | Boys | 19.9 / 22.2 | 19.6 / 21.8 | 20.0 / 22.5 |
|  | Girls | 20.1 / 22.0 | 19.7 / 21.9 | 20.0 / 22.8 |
| 10.5 | Boys | 20.4 / 22.8 | 20.1 / 22.3 | 20.5 / 23.0 |
|  | Girls | 20.8 / 22.6 | 20.4 / 22.5 | 20.5 / 23.2 |
| 11.0 | Boys | 20.9 / 23.3 | 20.6 / 22.8 | 21.0 / 23.5 |
|  | Girls | 21.5 / 23.3 | 21.0 / 23.0 | 21.2 / 23.8 |
| 11.5 | Boys | 21.3 / 23.8 | 21.1 / 23.2 | 21.5 / 24.0 |
|  | Girls | 22.2 / 24.0 | 21.5 / 23.5 | 22.0 / 24.5 |
| 12.0 | Boys | 21.8 / 24.2 | 21.5 / 23.8 | 21.2 / 24.5 |
|  | Girls | 22.8 / 24.8 | 22.0 / 24.0 | 22.0 / 25.0 |
| 12.5 | Boys | 22.3 / 24.7 | 21.8 / 24.2 | 22.0 / 25.0 |
|  | Girls | 23.2 / 25.4 | 22.2 / 24.5 | 22.5 / 25.5 |
| 13.0 | Boys | 22.8 / 25.2 | 22.2 / 24.8 | 22.5 / 25.5 |
|  | Girls | 23.5 / 25.8 | 22.5 / 25.0 | 23.0 / 26.0 |
| 13.5 | Boys | 23.3 / 25.7 | 22.6 / 25.3 | 23.0 / 26.0 |
|  | Girls | 23.8 / 26.0 | 22.8 / 25.5 | 23.5 / 26.5 |
| 14.0 | Boys | 23.8 / 26.2 | 22.8 / 25.8 | 23.5 / 26.5 |
|  | Girls | 23.8 / 26.3 | 23.0 / 25.8 | 24.0 / 27.0 |
| 14.5 | Boys | 24.3 / 26.7 | 23.2 / 26.2 | 23.8 / 27.0 |
|  | Girls | 23.9 / 26.6 | 23.3 / 26.2 | 24.5 / 27.5 |
| 15.0 | Boys | 24.7 / 27.2 | 23.5 / 26.5 | 24.2 / 27.5 |
|  | Girls | 23.9 / 26.8 | 23.5 / 26.5 | 24.8 / 27.8 |
| 15.5 | Boys | 25.2 / 27.6 | 23.8 / 26.8 | 24.5 / 27.8 |
|  | Girls | 23.9 / 27.0 | 23.7 / 26.8 | 25.0 / 28.0 |
| 16.0 | Boys | 25.6 / 28.0 | 24.2 / 27.2 | 25.0 / 28.0 |
|  | Girls | 24.0 / 27.2 | 24.0 / 27.2 | 25.0 / 28.0 |
| 16.5 | Boys | 26.0 / 28.4 | 24.4 / 27.5 | 25.3 / 28.5 |
|  | Girls | 24.0 / 27.6 | 24.2 / 27.5 | 25.5 / 28.5 |
| 17.0 | Boys | 26.4 / 28.8 | 24.6 / 27.8 | 25.8 / 29.0 |
|  | Girls | 24.0 / 27.8 | 24.5 / 27.8 | 25.5 / 28.5 |
| 17.5 | Boys | 26.8 / 29.2 | 24.8 / 28.0 | 26.0 / 29.5 |
|  | Girls | 24.0 / 28.0 | 24.8 / 28.0 | 25.8 / 29.0 |
| 18.0 | Boys | 27.2 / 29.6 | 25.0 / 30.0 | 25.0 / 30.0 |
|  | Girls | 24.0 / 28.0 | 25.0 / 30.0 | 25.0 / 30.0 |

**Supplement method 2.** Clinical parameters and instruments

| **Clinical Parameters** | **Abbreviation** | **Instrument** |
| --- | --- | --- |
| Alanine Aminotransferase | ALT | Beckman Coulter AU 5800 |
| Aspartate Aminotransferase | AST |  |
| **Gamma-Glutamyl Transferase** | GGT |  |
| Glucose | GLU |  |
| **Total Cholesterol** | **Chol** |  |
| **Triglycerides** | TG |  |
| **High-Density Lipoprotein** | HDL |  |
| **Low-Density Lipoprotein** | LDL |  |
| **Uric Acid** | UA |  |
| **Creatinine** | **CREA** |  |
| **Urea** | UREA |  |
| **Insulin** | INS | Roche e801 |
| **C-peptide** | C-P |  |
| Glycated Hemoglobin | HbA1c | Tosoh HLC-723G-11 |
| Systolic Blood Pressure | SBP | Omron HBP-1320 |
| Diastolic Blood Pressure‌ | DBP |  |
| Body Fat Percentage | BF | Inbody 770 |

After fasting for 8 hours, venous blood was collected by professionals. Then, the sample is centrifuged at 3,500 rpm for 15 minutes, and the supernatant is collected for blood biochemical testing and glucose metabolism testing. Whole blood samples were collected in tubes with anticoagulants for glycated hemoglobin testing.

The study measured blood pressure in the right upper arm of the subjects in a resting state using an upper arm electronic blood pressure monitor. Participants placed their elbow on the table, ensuring it was at the same level as the heart. The cuff was wrapped around the upper arm, with its lower edge positioned 2-3 cm above the elbow crease, and the midpoint of the cuff aligned with the heart. Participants remained calm and quiet during the measurement. Three measurements were taken, and the final data recorded was the average of the last two measurements.

Before conducting body composition measurements, participants are required to empty their bladders and bowels as much as possible to minimize interference from excess weight. Remove heavy clothing, shoes, socks, and metal objects. Stand barefoot on the device's electrode plates, ensuring that the soles of the feet are in full contact with the electrodes. Keep the arms naturally relaxed at the sides, without touching the torso. Grip the handle electrodes firmly with both hands, align your thumbs with the sensors, and keep your arms separated from your torso at an angle of 15° to 30° during the body composition analysis.

**Supplement method 3.** Saliva and fecal sample collection

Saliva and fecal samples were collected by following the standard sampling procedures of the Human Microbiome Project("Human Microbiome Project Standard Operating Procedures "). Before stool sample collection, participants were asked to empty their bladders to avoid mixing urine during the sampling process. A sterile fecal collection box was positioned in the middle back of the toilet seat. After defecation, wearing gloves and a mask, a sterile cotton swab was used to dislodge the feces in the collection box, and a sampling spoon was used to scoop 1 spoonful of sample from the inside of the middle of the feces, with a collection volume of approximately 2 grams, into the preservation tube. Saliva sampling was performed in the morning, and participants were asked to refrain from eating, brushing their teeth, or using mouthwash. For non-irritant saliva collection, participants were asked to naturally secrete saliva and keep it in their mouths for 1 minute, and then use a sterile disposable collection tube to collect saliva in a volume of approximately 2 mL. After collection, the cap of the tube was quickly tightened, and the tube was labeled with the sample information. The samples were placed in dry ice for temporary storage and transferred to a -80°C deep freezer for freezing within 2 hours.

Reference:

Human Microbiome Project Standard Operating Procedures Retrieved from https://www.hmpdacc.org/hmp/resources/

**Supplement method 4.** Random forest analysis using 10-times repeated 10-fold cross-validation detailed parameter

| Parameter | Value |
| --- | --- |
| Number of trees (ntree) | 500 |
| Cross-validation scheme | 10-times repeated 10-fold cross-validation (10 × 10 CV) |
| Train/validation split per fold | ~122-123 samples for training, ~13-14 for validation |
| Train/test split | No explicit separate test set; 10×10 CV serves as internal validation |
| Hyperparameter tuning | Grid search over mtry = 3, 5, 7, 9, 11, 13, 15 |
| Optimal mtry | 15 (selected by highest ROC AUC) |

Random forest model performance metrics (based on 10×10 repeated cross-validation)

| Metric | Value |
| --- | --- |
| ROC AUC | 0.7419 |
| Sensitivity | 0.7190 |
| Specificity | 0.6410 |
| Accuracy | 0.660 |
| Precision | 0.640 |
| F1 Score | 0.677 |

**Supplement method 5.** R package versions used in this study (R 4.2.1)

| Category | Package | Version | Primary Function |
| --- | --- | --- | --- |
| **Data Import** | readxl | 1.4.5 | Read Excel files (.xls, .xlsx) |
|  | openxlsx | 4.2.5.2 | Read/write Excel files with formatting |
| **Data Manipulation** | dplyr | 1.1.4 | Data transformation (filter, mutate, summarise, join) |
|  | tidyr | 1.3.0 | Data tidying (pivot, separate, unite) |
|  | reshape2 | 1.4.4 | Data reshaping (melt, dcast) |
|  | stringr | 1.5.0 | String manipulation |
| **Statistical Analysis** | rstatix | 0.7.2 | Pipe-friendly statistical tests (t-test, ANOVA, Wilcoxon) |
|  | Hmisc | 5.1-0 | Correlation matrices (rcorr), data description |
|  | CBCgrps | 2.8.2 | Clinical baseline characteristics table (Table 1) |
|  | vegan | 2.6-4 | Ecological/community analysis (diversity indices, PERMANOVA, PCA, NMDS) |
|  | cluster | 2.1.4 | Clustering algorithms (PAM, hierarchical) |
| **Machine Learning** | RandomForest | 4.7-1.2 | Random forest classification/regression, feature importance |
|  | caret | 7.0-1 | Classification And Regression Training |
| **Visualization** | ggplot2 | 4.0.1 | Core plotting system (grammar of graphics) |
|  | ggpubr | 0.5.0 | Publication-ready plots with automatic statistical tests |
|  | gghalves | 0.1.4 | Half-violin/half-box plots |
|  | pheatmap | 1.0.12 | Heatmap with clustering |
|  | factoextra | 1.0.7 | Extract and visualize multivariate results (PCA, clustering) |
|  | gridExtra | 2.3 | Arrange multiple ggplot2 plots |

**Supplement Table 1.** Relative abundance of microbiota at the phylum level.

| **Oral Phylum** | **NW** | **OB** | **FDR** |  | **Gut Phylum** | **NW** | **OB** | **FDR** |  |
| --- | --- | --- | --- | --- | --- | --- | --- | --- | --- |
| Firmicutes | 39.18 | 34.2 | * |  | Firmicutes | 48.17 | 46.12 | ns |  |
| Bacteroidetes | 25.9 | 27.45 | ns |  | Bacteroidetes | 39.16 | 38.9 | ns |  |
| Proteobacteria | 24.36 | 25.81 | ns |  | Proteobacteria | 6.92 | 9.87 | ns |  |
| Fusobacteria | 4.53 | 5.73 | ns |  | Actinobacteria | 5.13 | 4.42 | ns |  |
| Actinobacteria | 4.16 | 4.43 | ns |  | Fusobacteria | 0.23 | 0.33 | ns |  |
| Saccharibacteria | 1.4 | 1.68 | ns |  | Verrucomicrobia | 0.2 | 0.14 | ns |  |
| Spirochaetae | 0.1 | 0.09 | ns |  | Tenericutes | 0.12 | 0.13 | ns |  |
| Gracilibacteria | 0.05 | 0.12 | ns |  | Saccharibacteria | 0.01 | 0.04 | ns |  |
| Tenericutes | 0.02 | 0.03 | ns |  | Lentisphaerae | 0.03 | 0.01 | ns |  |
| Synergistetes | 0.01 | 0.02 | ns |  | Synergistetes | 0.01 | 0.01 | ns |  |
| Deinococcus-Thermus | 7.57×10^-04^ | 3.44×10^-03^ | ns |  | Cyanobacteria | 0 | 8.91×10^-03^ | ns |  |
| Chloroflexi | 3.30×10^-03^ | 1.03×10^-04^ | ns |  | Deinococcus-Thermus | 9.33×10^-04^ | 0 | ns |  |
| Cyanobacteria | 9.63×10^-04^ | 3.10×10^-04^ | ns |  | Spirochaetae | 2.97×10^-04^ | 2.54×10^-04^ | ns |  |
| Verrucomicrobia | 3.44×10^-04^ | 5.50×10^-04^ | ns |  | Acidobacteria | 4.24×10^-05^ | 4.24×10^-05^ | ns |  |
| Planctomycetes | 2.41×10^-04^ | 0 | ns |  | Gracilibacteria | 8.48×10^-05^ | 0 | ns |  |
| Ignavibacteriae | 1.03×10^-04^ | 0 | ns |  | Parcubacteria | 0 | 4.24×10^-05^ | ns |  |
| Gemmatimonadetes | 6.88×10^-05^ | 0 | ns |  | Others | 0.01 | 0.01 | ns |  |
| Chlorobi | 0 | 3.44×10^-05^ | ns |  |  |  |  |  |  |
| Lentisphaerae | 3.44×10^-05^ | 0 | ns |  |  |  |  |  | |
| Acidobacteria | 3.44×10^-05^ | 0 | ns |  |  |  |  |  | |
| Others | 0.29 | 0.42 | ns |  |  |  |  |  | |

ns, non-significant; *FDR < 0.05, ** FDR < 0.01, *** FDR < 0.001.

**Supplement Table 2.** Relative abundance of microbiota at the genus level.

| **Oral Genus** | **NW** | **OB** | **FDR** |  | **Gut Genus** | **NW** | **OB** | **FDR** |
| --- | --- | --- | --- | --- | --- | --- | --- | --- |
| *Streptococcus* | 14.7 | 20.88 | *** |  | *Bacteroides* | 30.5 | 29.3 | ns |
| *Prevotella* | 15.77 | 14.65 | ns |  | *Faecalibacterium* | 8.29 | 7.4 | ns |
| *Neisseria* | 13.6 | 9.53 | ns |  | *Bifidobacterium* | 3.95 | 3.3 | ns |
| *Haemophilus* | 8.61 | 9.88 | ns |  | *Lachnospira* | 2.69 | 3.98 | ns |
| *Veillonella* | 9.7 | 8.21 | ns |  | *Prevotella* | 2.24 | 4.05 | ns |
| *Alloprevotella* | 6.19 | 6.34 | ns |  | *Parasutterella* | 2.41 | 3.69 | ns |
| *Porphyromonas* | 4.05 | 3.3 | ns |  | *Megamonas* | 3.75 | 1.76 | ns |
| *Fusobacterium* | 3.47 | 2.72 | ns |  | *Parabacteroides* | 2.6 | 2.51 | ns |
| *Granulicatella* | 2.78 | 3.02 | ns |  | *Ruminococcus* | 2.96 | 2.07 | ns |
| *Rothia* | 2.65 | 2.53 | ns |  | *Roseburia* | 1.95 | 2.84 | ns |
| *Leptotrichia* | 2.08 | 1.67 | ns |  | *Subdoligranulum* | 2.17 | 2.45 | ns |
| *Aggregatibacter* | 1.38 | 1.22 | ns |  | *Alistipes* | 2.11 | 2.03 | ns |
| *Gemella* | 0.96 | 1.48 | * |  | *Lachnoclostridium* | 1.52 | 1.92 | ns |
| *Actinobacillus* | 0.53 | 1.38 | * |  | *Escherichia* | 1.67 | 1.51 | ns |
| *Actinomyces* | 0.89 | 0.79 | ns |  | *Dialister* | 1.18 | 1.79 | ns |
| *Lachnoanaerobaculum* | 0.77 | 0.73 | ns |  | *Blautia* | 1.5 | 1.37 | ns |
| *Capnocytophaga* | 0.69 | 0.56 | ns |  | *Phascolarctobacterium* | 1.39 | 1.12 | ns |
| *Oribacterium* | 0.65 | 0.58 | ns |  | *Collinsella* | 1.08 | 0.78 | ns |
| *Megasphaera* | 0.71 | 0.49 | ns |  | *Sutterella* | 0.61 | 1.05 | ns |
| *Peptostreptococcus* | 0.71 | 0.45 | * |  | *Ruminiclostridium* | 0.95 | 0.69 | ns |
| *Others* | 9.12 | 9.60 | ns |  | *Others* | 24.48 | 24.39 | ns |

ns, non-significant; *FDR < 0.05, ** FDR < 0.01, *** FDR < 0.001.

**Supplement Table 3.** Mean Decrease Gini for co-present genera in the oral and gut

|  | NW | OB | Mean Decrease Gini |
| --- | --- | --- | --- |
| *s_Bifidobacterium* | 4.585 | 6.195 | 2.456 |
| *s_Streptococcus* | 5.080 | 4.650 | 2.111 |
| *s_Neisseria* | 5.613 | 4.726 | 1.864 |
| *s_Gemella* | 2.553 | 2.302 | 1.382 |
| *s_Peptostreptococcus* | 3.145 | 3.879 | 1.289 |
| *s_Dialister* | 1.115 | 0.870 | 1.164 |
| *s_Parvimonas* | 2.878 | 4.217 | 1.104 |
| *s_Escherichia* | -0.448 | 3.000 | 1.092 |
| *s_Oribacterium* | 1.237 | 3.022 | 1.088 |
| *s_Actinobacillus* | 1.624 | 3.070 | 1.064 |
| *f_Anaerostipes* | 0.289 | 0.796 | 1.017 |
| *s_Bacteroides* | 1.193 | 1.037 | 0.949 |
| *s_Selenomonas* | 1.959 | 0.031 | 0.940 |
| *s_Parasutterella* | 1.098 | 1.991 | 0.908 |
| *s_Porphyromonas* | 0.252 | 1.413 | 0.892 |
| *f_Bifidobacterium* | -1.276 | -1.327 | 0.889 |
| *f_Roseburia* | 0.948 | 0.674 | 0.864 |
| *s_Lautropia* | 2.470 | -0.353 | 0.856 |
| *f_Lachnospira* | 0.738 | -0.257 | 0.855 |
| *f_Paraprevotella* | 2.392 | 2.442 | 0.774 |
| *f_Parasutterella* | 0.992 | 0.208 | 0.766 |
| *s_Veillonella* | 0.577 | 0.442 | 0.751 |
| *s_Campylobacter* | 1.260 | 2.145 | 0.737 |
| *s_Parabacteroides* | 2.078 | 2.528 | 0.726 |
| *s_Subdoligranulum* | 0.673 | 0.668 | 0.719 |
| *s_Faecalibacterium* | 2.564 | 2.527 | 0.703 |
| *s_unclassified* | -0.079 | 1.554 | 0.696 |
| *s_Peptococcus* | -0.438 | -0.643 | 0.621 |
| *s_Abiotrophia* | 0.963 | 0.270 | 0.611 |
| *s_Aggregatibacter* | 1.038 | -1.476 | 0.605 |
| *f_Butyricicoccus* | 1.021 | -0.234 | 0.599 |
| *s_Prevotella* | 1.437 | 0.731 | 0.585 |
| *s_Actinomyces* | 0.399 | -0.646 | 0.573 |
| *s_Ruminococcus* | 1.754 | 0.886 | 0.572 |
| *f_Fusicatenibacter* | -1.930 | -0.980 | 0.561 |
| *s_Fusobacterium* | -0.602 | 0.247 | 0.558 |
| *s_Solobacterium* | 1.566 | -2.230 | 0.557 |
| *s_Leptotrichia* | -1.868 | -1.360 | 0.556 |
| *s_Lachnoanaerobaculum* | 0.585 | 1.017 | 0.551 |
| *f_Faecalibacterium* | 1.001 | -1.112 | 0.551 |
| *f_Alistipes* | 0.320 | -1.235 | 0.546 |
| *s_Enterobacter* | 3.914 | 4.369 | 0.540 |
| *s_Corynebacterium* | -1.566 | -1.155 | 0.531 |
| *s_Megasphaera* | 1.878 | 1.867 | 0.529 |
| *s_Haemophilus* | -1.255 | 0.904 | 0.522 |
| *f_Porphyromonas* | 2.957 | 0.689 | 0.520 |
| *f_Ruminococcus* | 0.024 | -0.775 | 0.518 |
| *s_Lachnoclostridium* | 1.362 | 3.527 | 0.516 |
| *s_Rothia* | -0.175 | -0.064 | 0.516 |
| *f_Subdoligranulum* | 1.219 | -0.213 | 0.516 |
| *f_Haemophilus* | -1.148 | 0.198 | 0.516 |
| *f_Veillonella* | 0.665 | 1.327 | 0.516 |
| *f_Lachnoclostridium* | -1.410 | -0.124 | 0.512 |
| *f_Tyzzerella* | 0.795 | -2.299 | 0.509 |
| *s_Bergeyella* | -0.513 | -1.868 | 0.507 |
| *f_Sutterella* | -1.013 | -0.476 | 0.505 |
| *f_Dialister* | 1.337 | -0.718 | 0.504 |
| *f_Phascolarctobacterium* | -1.170 | -0.740 | 0.494 |
| *s_Mogibacterium* | -0.735 | 1.391 | 0.474 |
| *f_Klebsiella* | 0.228 | 1.414 | 0.473 |
| *s_Atopobium* | -1.427 | 0.263 | 0.468 |
| *s_Alloprevotella* | -0.162 | -0.658 | 0.467 |
| *s_Capnocytophaga* | -0.669 | -1.222 | 0.465 |
| *f_Ruminiclostridium* | -0.488 | 0.594 | 0.462 |
| *f_Escherichia* | 0.073 | 0.791 | 0.454 |
| *f_Bacteroides* | 0.784 | 0.234 | 0.451 |
| *s_Lachnospira* | 0.723 | 0.486 | 0.451 |
| *f_Blautia* | -0.558 | -0.876 | 0.448 |
| *s_Sutterella* | -0.236 | 0.800 | 0.441 |
| *s_Moraxella* | -1.645 | 3.851 | 0.435 |
| *f_unclassified* | 0.484 | -0.052 | 0.429 |
| *s_Granulicatella* | 1.006 | 2.336 | 0.427 |
| *s_Phascolarctobacterium* | 0.228 | 2.556 | 0.426 |
| *f_Parabacteroides* | 1.270 | -0.772 | 0.420 |
| *f_Granulicatella* | 0.211 | 1.272 | 0.415 |
| *f_Coprococcus* | -0.961 | 0.837 | 0.413 |
| *f_Actinomyces* | -0.228 | 1.679 | 0.410 |
| *f_Enterobacter* | -0.882 | 0.750 | 0.410 |
| *s_Roseburia* | 0.182 | 2.917 | 0.406 |
| *f_Collinsella* | -1.886 | -0.127 | 0.406 |
| *f_Prevotella* | 1.179 | 1.241 | 0.395 |
| *f_Selenomonas* | 2.989 | 0.438 | 0.395 |
| *f_Alloprevotella* | 1.340 | 0.746 | 0.392 |
| *s_Acinetobacter* | 0.443 | 0.158 | 0.377 |
| *s_Massilia* | -2.727 | 0.643 | 0.370 |
| *f_Neisseria* | -0.368 | -2.239 | 0.368 |
| *s_Butyricicoccus* | 1.058 | 2.929 | 0.366 |
| *f_Bilophila* | -1.137 | 0.638 | 0.366 |
| *f_Streptococcus* | -1.012 | -0.774 | 0.362 |
| *f_Odoribacter* | 0.359 | -2.073 | 0.354 |
| *s_Bilophila* | -0.816 | 0.371 | 0.348 |
| *f_Flavonifractor* | -2.192 | -1.309 | 0.317 |
| *f_Barnesiella* | 1.165 | -1.764 | 0.311 |
| *f_Gemella* | 0.103 | -0.982 | 0.309 |
| *f_Peptostreptococcus* | 1.896 | 1.820 | 0.301 |
| *f_Fusobacterium* | -0.735 | -1.193 | 0.283 |
| *f_Megamonas* | -1.203 | -0.624 | 0.275 |
| *f_Peptococcus* | 0.058 | -0.319 | 0.275 |
| *f_Megasphaera* | -1.106 | -0.828 | 0.258 |
| *f_Campylobacter* | -0.117 | 0.243 | 0.250 |
| *f_Rothia* | -0.779 | -0.543 | 0.235 |
| *f_Aggregatibacter* | -0.849 | 0.132 | 0.233 |
| *s_Megamonas* | -0.687 | 1.290 | 0.232 |
| *f_Acinetobacter* | 0.730 | 1.448 | 0.226 |
| *f_Actinobacillus* | -0.292 | -1.575 | 0.223 |
| *f_Lactobacillus* | -1.174 | 0.696 | 0.220 |
| *f_Bacillus* | -1.989 | 1.748 | 0.217 |
| *f_Atopobium* | 0.871 | 1.219 | 0.214 |
| *s_Blautia* | -0.748 | 1.607 | 0.210 |
| *f_Pseudomonas* | -1.765 | 0.817 | 0.210 |
| *f_Leptotrichia* | 0.869 | 0.332 | 0.195 |
| *s_Alistipes* | -0.555 | 0.378 | 0.194 |
| *f_Solobacterium* | -1.092 | -0.643 | 0.192 |
| *s_Pseudomonas* | -0.425 | 0.339 | 0.186 |
| *s_Fusicatenibacter* | 0.555 | -0.009 | 0.175 |
| *s_Klebsiella* | 0.573 | 0.236 | 0.169 |
| *f_Abiotrophia* | 0.719 | 2.389 | 0.165 |
| *s_Tyzzerella* | 0.709 | 0.044 | 0.147 |
| *s_Ruminiclostridium* | -0.622 | -0.313 | 0.144 |
| *f_Massilia* | -1.525 | 1.273 | 0.140 |
| *s_Odoribacter* | -0.631 | 1.038 | 0.130 |
| *s_Barnesiella* | 2.611 | 1.155 | 0.123 |
| *s_Collinsella* | -1.451 | 0.480 | 0.122 |
| *s_Paraprevotella* | 1.897 | 2.103 | 0.120 |
| *f_Mogibacterium* | 0.811 | -1.149 | 0.117 |
| *f_Lachnoanaerobaculum* | -2.006 | 0.251 | 0.117 |
| *s_Lactobacillus* | 0.366 | 0.404 | 0.116 |
| *f_Corynebacterium* | 1.975 | 0.400 | 0.112 |
| *s_Anaerostipes* | -1.095 | 1.088 | 0.101 |
| *s_Bacillus* | -1.000 | -1.054 | 0.098 |
| *f_Capnocytophaga* | -1.346 | -0.965 | 0.096 |
| *s_Flavonifractor* | -1.768 | -0.701 | 0.093 |
| *f_Parvimonas* | -0.372 | -0.333 | 0.093 |
| *f_Oribacterium* | 0.527 | 1.411 | 0.079 |
| *s_Coprococcus* | -0.774 | -0.553 | 0.079 |
| *f_Bergeyella* | 0.005 | 1.411 | 0.073 |
| *f_Lautropia* | 1.662 | -2.005 | 0.060 |
| *f_Moraxella* | 0.091 | 0.059 | 0.049 |

NW, normal weight; OB, obesity.

s_, saliva; f_, feces.

**A	B**

**C	D**

**Supplement Figure 1.**

(A and C) Rarefaction curves for oral (A) and gut (C) microbiota.

(B and D) Alpha diversity index of oral (B) and gut (D) microbiota. Boxplots show medians; the lower and upper hinges correspond to the first and third quartiles (the 25th and 75th percentiles); whiskers extend to the largest and smallest values at 1.5*IQR (interquartile range) from the hinge. Intergroup comparisons were performed using the Wilcoxon test.

NW, normal weight. OB, obesity. **P* < 0.05, ** *P* < 0.01, *** *P* < 0.001.

**A	B**

**C	D**

**Supplement figure 2.**

(A and C) Stacked bar plots showing relative abundances of oral (A) microbiota and gut (C) microbiota at phylum level in obesity or normal weight children.

(B and D) Stacked bar plots showing relative abundances of oral (B) microbiota and gut (D) microbiota at phylum level in obesity or normal weight children.

NW, normal weight. OB, obesity.

**A	B**

**Supplement figure 3.**

Linear discriminant analysis (LDA) effect size showed a list of specific oral (A) and gut (B) microbiota that enable discrimination between obesity and normal weight children.

p, phylum; c, class; o, order; f, family; g, genus. NW, normal weight. OB, obesity.

**A	B**

**Supplement figure 4.**

The Kyoto Encyclopedia of Genes and Genomes (KEGG) analysis of microbiota associated with children obesity. Comparison of KEGG pathways at level 1 in the oral (A) and gut (B) microbiota. NW, normal weight; OB, obesity; SE, Standard Error. **P* < 0.05, ** *P* < 0.01, *** *P* < 0.001

**Supplement figure 5.**

Principal coordinate analysis (PCoA) based on Bray-Curtis dissimilarities, color-coded by location with purple representing oral microbiota and orange representing gut microbiota.

**A	B**

**Supplement figure 6.**

Heatmaps of sparse correlations for compositional data among relative abundances of the top 30 shared genera within oral (A) or gut (B) microbiota.

\# FDR< 0.1, * FDR< 0.05, ** FDR< 0.01, *** FDR < 0.001.

**Supplement figure 7.**

Prevalence and relative abundances of the top 30 shared genera in obesity and normal weight children. NW, normal weight. OB, obesity.

|  | AUC1 | AUC2 | Z / D | P |
| --- | --- | --- | --- | --- |
| combined vs oral-only | 0.73 | 0.78 | D = -8.33 | < 0.001 |
| combined vs gut-only | 0.73 | 0.55 | D = 22.81 | < 0.001 |
| oral -only vs gut -only | 0.78 | 0.55 | Z = 31.78 | < 0.001 |

**Supplement figure 8.** ROC curves of random forest models using combined, oral-only, and gut-only co-present genera.

**Supplement figure 9.**

Out-of-Bag (OOB) error convergence curve of the random forest model.
